# A lightweight convolutional neural network for recognition of severity stages of maydis leaf blight disease of maize

**DOI:** 10.3389/fpls.2022.1077568

**Published:** 2022-12-19

**Authors:** Md. Ashraful Haque, Sudeep Marwaha, Alka Arora, Chandan Kumar Deb, Tanuj Misra, Sapna Nigam, Karambir Singh Hooda

**Affiliations:** ^1^ Division of Computer Applications, Indian Council of Agriculture Research (ICAR)-Indian Agricultural Statistics Research Institute, New Delhi, India; ^2^ Department of Computer Science, Rani Lakshmi Bai Central Agricultural University, Jhansi, India; ^3^ Division of Germplasm Evaluation, Indian Council of Agriculture Research (ICAR)-National Bureau of Plant Genetic Resources, New Delhi, India

**Keywords:** maydis leaf blight disease, maize crop, disease severity stages, MDSD image database, convolutional neural network, inception module

## Abstract

Maydis leaf blight (MLB) of maize (*Zea Mays L.*), a serious fungal disease, is capable of causing up to 70% damage to the crop under severe conditions. Severity of diseases is considered as one of the important factors for proper crop management and overall crop yield. Therefore, it is quite essential to identify the disease at the earliest possible stage to overcome the yield loss. In this study, we created an image database of maize crop, MDSD (Maydis leaf blight Disease Severity Dataset), containing 1,760 digital images of MLB disease, collected from different agricultural fields and categorized into four groups viz. healthy, low, medium and high severity stages. Next, we proposed a lightweight convolutional neural network (CNN) to identify the severity stages of MLB disease. The proposed network is a simple CNN framework augmented with two modified *Inception* modules, making it a lightweight and efficient multi-scale feature extractor. The proposed network reported approx. 99.13% classification accuracy with the f1-score of 98.97% on the test images of MDSD. Furthermore, the class-wise accuracy levels were 100% for healthy samples, 98% for low severity samples and 99% for the medium and high severity samples. In addition to that, our network significantly outperforms the popular pretrained models, viz. VGG16, VGG19, InceptionV3, ResNet50, Xception, MobileNetV2, DenseNet121 and NASNetMobile for the MDSD image database. The experimental findings revealed that our proposed lightweight network is excellent in identifying the images of severity stages of MLB disease despite complicated background conditions.

## 1 Introduction

In India, maize (*Zea Mays* L.) is the third most important cereal grain crop. The maize crop is being grown in Kharif and rabi seasons across the country (Kaur et al., 2020). It is considered as the ‘Queen of Cereals’ due to its multiple use cases, such as staple food for human beings, feed-fodder for livestock animals, raw materials for several processed foods, industrial products, a rich source of starch and so on. As per the reports, around 31.65 mt of maize was produced across the country during 2020-2021 (ICAR-IIMR, 2021). Every year, around 13.2% of the total crop yield is damaged due to the attack of several disease-causing pathogens (Aggarwal et al., 2021). Among several diseases, Maydis leaf blight or MLB (aka Southern corn leaf Blight) is a serious fungal disease across maize-growing regions of India. Generally, the country’s warm and humid climatic condition is extremely favorable for the disease development (Malik et al., 2018). The MLB disease is caused by *Bipolaris maydis (Nisik. & Miyake)* Shoemaker 1959 fungus. In the early stages, its symptoms appear as small and oval to diamond-shaped, necrotic to brown-colored lesions on the leaf surfaces. These lesions get elongated as the disease progresses (Aggarwal et al., 2021). It is reported that this disease alone is capable of causing damage approx. 70% of the total crop yield in severe conditions (Hooda et al., 2018). The severity of diseases is an important parameter that measures the intensity level of disease symptoms in the affected portion of the crop and is crucial for disease management too (Hooda et al., 2018). Therefore, our first and foremost aim must be to identify and control the disease at the earliest possible stage of severity to minimize the risk of potential yield loss of maize crop. However, the conventional approach for identifying the severity stages involves visual observations and laboratory analysis. But the fact is, these approaches require highly trained and experienced personnel, which makes them practically infeasible many times. Hence, there is a much need for a precise, quick, cost-effective and automated approach to identify the disease severity stages in the field conditions.

In recent years, several computer vision techniques have been applied to several challenging agricultural problems (Kamilaris and Prenafeta-Boldú, 2018). In this connection, the convolutional neural networks (aka CNNs) are considered as the benchmark for different image-based problem identification in the agriculture domain. The CNN approaches have eased the image recognition process by automatically extracting the features from the images as compared to the hand-engineered feature extractions in the traditional machine learning approaches (LeCun et al., 2015). In case of diagnosis of diseases as well as their severity stages, CNNs have shown significantly better results than the traditional image processing and machine learning techniques. In this context, a very limited number of works have been reported to diagnose disease severity stages in maize crop using in-field images. Therefore, we proposed a novel lightweight CNN network for identifying the severity stages of MLB disease in maize crop. This network would be a practical and viable solution for the farm community of the country. The main contributions of this study are provided below:

- Created an image database known as MDSD (Maydis leaf blight Disease Severity Dataset) containing digital images of maize leaves infected with MLB disease covering all severity stages. The images of MDSD were collected in non-destructive manner with natural field backgrounds from different agricultural fields.
- Proposed a lightweight and efficient convolutional neural network (CNN) model augmented with modified inception modules. The proposed network is trained and validated on the images of the MDSD database for automatic identification of severity stages of MLB disease.
- To evaluate the effectiveness of the proposed network, we conducted a comparative analysis of the prediction performance between the proposed model and a few popular state-of-the-art pretrained networks.

This article is organized into six sections. Section 1 (present section) highlights the importance of maize crop, the devastating effect of MLB diseases, constraints of the conventional approaches of disease recognition and management, importance of computer vision-based technologies *etc.*: Section 2 explores and briefly discusses the related works relevant to the present study, Section 3 explains the materials and methodologies used to carry out the current study; Section 4 reports and discusses the experimental results and finding of the study; Section 5 presents the ablation studies; and Section 6 concludes the whole study highlighting the impact and crucial finding and aligns the future perspective of this study.

## 2 Related work

In this section, we will briefly discuss the methodologies proposed by research works from across the globe for recognizing diseases as well their severity stages. In recent years, deep learning-based techniques are gaining momentum for identifying diseases of several crops. Several authors like Mohanty et al. (2016); Sladojevic et al. (2016); Ferentinos (2018); Barbedo (2019) and Atila et al. (2021) focused on identifying the diseases of crops at once by applying variety of deep learning models such as state-of-the-art networks, transfer learning models, custom defined models, hybrid CNN models and many more. These works targeted identifying diseases of multiple crops by a single deep learning model. Whereas most of the reported works aimed at crop-specific disease identification problems such as for Rice crop (Lu et al., 2017; Chen et al., 2020; Rahman et al., 2020), Wheat crop (Lu et al., 2017; Picon et al., 2019; Nigam et al., 2021), Tomato crop (Fuentes et al., 2018; Zhang et al., 2018), Maize crop (DeChant et al., 2017; Priyadharshini et al., 2019; Lv et al., 2020; Haque et al., 2021; Haque et al., 2022a; Haque et al., 2022b), etc. The experimental findings of these research works reported significant results by employing several types of CNN-based networks to identify the diseases using color images. Some of these works used lab-based images of crop-diseases such as plant village for their model development, while some has used in-field images

Nowadays, the identification of severity stages of diseases has also attracted the attention of researchers. Significant works have been carried out to identify the disease severity stages using digital images. Wang et al. (2017) applied transfer learning of popular deep CNN models to diagnose disease severity in apple plants and obtained more than 94% classification accuracy on the test dataset. They used publicly available images and assessed them into 4 categories of severity stages for their experiment. Liang et al. (2019) proposed a robust approach for disease diagnosis and disease severity estimation of several crops using deep learning models. Verma et al. (2020) worked on tomato late blight disease and Prabhakar et al. (2020) worked on estimating the severity stages of tomato early blight disease using Deep CNN models. Recently, Sibiya and Sumbwanyambe (2021) used Deep CNN models to classify images of common rust disease of maize crop into four classes of severity levels. They applied fuzzy logic-based techniques to automatically categorize the diseased images into severity categories. Nigam et al. (2021) classified the stem rust disease of wheat crop into four severity categories using deep convolutional neural networks. Chen et al. (2021) worked on estimating the severity of the rice bacterial leaf streak disease using a segmentation-based approach. Wang et al. (2021) proposed an image-segmentation-based approach by integrating a deep CNN model to recognize severity stages of downy mildew, powdery mildew and cucumber viral diseases of cucumber crops. Ji and Wu (2022) proposed fuzzy logic integrated deep learning model for detecting the severity levels of grape black measles disease. Liu et al. (2022) developed a two-stage CNN model for diagnosing the severity of Alternaria leaf blotch disease of the Apple plant. A summary of the previous works is provided in **Table 1**.

**Table 1:** A brief summary of related works.

Authors	Work done	Dataset	Approach
Wang et al. (2017)	Diagnosis of disease severity in Apple plant	Plant Village dataset	Transfer learning approach
Liang et al. (2019)	Diagnosis of diseases and their severity levels of several crops	Own dataset	Custom CNN based on ResNet50 and ShuffleNet models
Verma et al. (2020)	Identification of severity levels of tomato late blight disease	Plant Village	Transfer learning approach
Prabhakar et al. (2020)	Detection of severity levels of tomato early blight disease	Plant Village	Pre-trained ResNet101 models
Sibiya and Sumbwanyambe (2021)	Classification of common rust disease of maize into four severity levels	PlantVillage dataset	OTSU threshold-segmentation method
Nigam et al. (2021)	Estimation of severity of stem rust disease of wheat	Own dataset	Custom CNN network
Chen et al. (2021)	Estimation severity of the Rice bacterial leaf streak disease	Own dataset	Segmentation-based CNN approach
Wang et al. (2021)	Recognition of severity stages of downy mildew, powdery mildew and cucumber viral diseases of cucumber	Own dataset	Image-segmentation-based CNN model
Ji and Wu (2022)	Detection of severity levels of black measles disease of grape	Own Dataset	Fuzzy logic integrated Deep learning approach
Liu et al. (2022)	Diagnosis of severity levels of Alternaria leaf blotch disease of Apple plant	Own dataset	Custom CNN model

## 3 Materials and methods

### 3.1 Flow of the proposed approach

**Figure 1:**
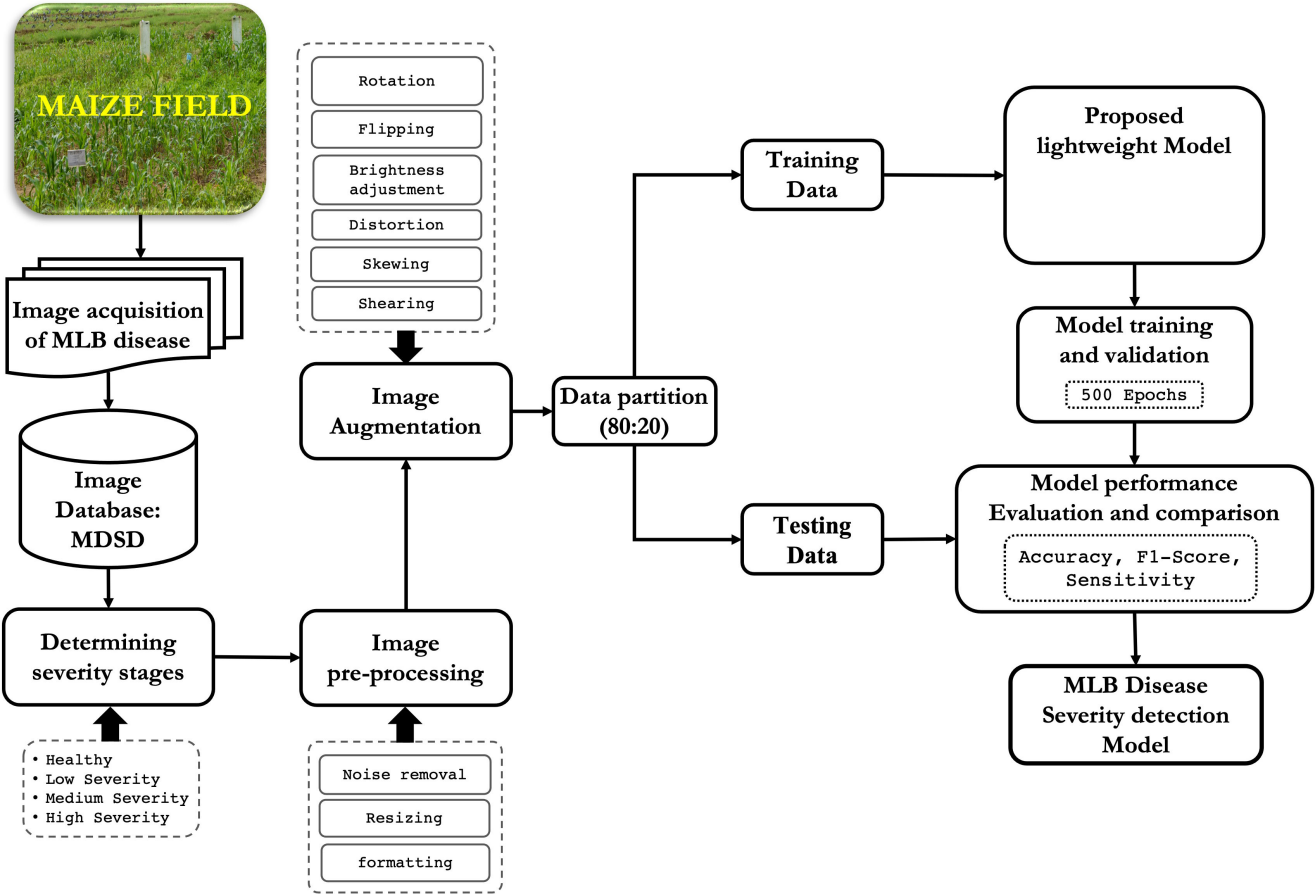
Overall framework of the proposed approach for recognition of severity stages of MLB disease.

The workflow of the proposed disease severity identification approach is depicted graphically in **Figure 1**. First, digital images of MLB disease of maize crop were captured from the fields and MDSD image database was created. Next, images were labelled into respective severity categories based on domain experts’ observations and saved into respective folders in the storage disk. Then, images were pre-processed and augmented to increase the training dataset; After that, the whole image dataset was split into two categories viz. training and testing sets and the proposed CNN model was trained and validated. Finally, based on the performance evaluation, the MLB disease-severity identification model was finalized and its architecture was saved on the disk. Detailed illustrations of these phases are discussed in the following sections.

### 3.2 Image acquisition

In this study, we created an image database known as MDSD containing digital images of maize leaves affected with MLB disease. The images were collected in a non-destructive manner from several agricultural plots located at Bidhan Chandra Krishi Visvavidyalaya, Kalyani (22.9920° N, 88.4495° E) and ICAR-Indian Agricultural Research Institute, New Delhi (28.6331° N, 77.1525° E) during 2018-2020. Digital cameras (Nikon D3500 W/AF) and smartphones (Redmi Y2 and Asus Max Pro M1) were used for capturing the images under normal daylight conditions. We collected the images of MLB disease by focusing the camera lens on the symptomatic portions of leaves starting from the disease incidence stage to the highest severity stage with complex field backgrounds.

### 3.3 Disease severity stages

**Figure 2:**
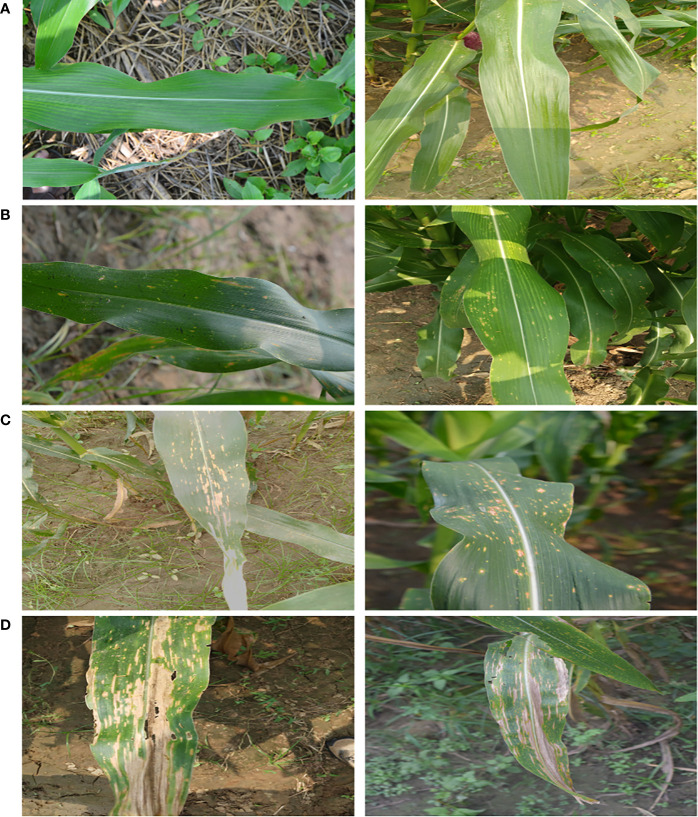
Sample images of MLB disease of maize crop grouped into four categories **(A)** Healthy **(B)** Low Severity **(C)** Medium Severity and **(D)** High Severity.

The images of MLB disease were thoroughly verified and categorized into four groups based on their symptomatic characteristics viz. healthy (no symptoms), low severity, medium severity and high severity stages as provided in **Table 2**. The categorization into the severity groups was done under the strict supervision of subject matter specialists (domain experts) of maize pathology at ICAR-IIMR, Ludhiana, India. Sample images of each category of MLB disease are shown in **Figure 2**.

**Table 2:** Categorization and summary of images of MDSD database.

Category	Characteristics	Original	Synthetic	Total
**Healthy**	No disease symptoms	511	3066	3577
**Low severity**	Disease symptoms cover <25% of the total leaf area	389	3112	3501
**Medium severity**	Disease symptoms cover 25-50% of the total leaf area	621	3105	3726
**High severity**	Disease symptoms cover > 50% of the total leaf area	239	3346	3585
	Total	1,760	**12629**	**14389**

### 3.4 Image pre-processing

Prior to training process, slight pre-processing of the raw images was required for better modelling. At first, unwanted images like duplicate, noisy, out-of-focus, blurred images were discarded from the raw images. After that, images were resized to 256 × 256 pixel size by keeping hardware system constraints in mind and for better interpretation by the proposed model.

### 3.5 Image augmentation

In order to increase the number of images for model training, synthetic images were generated and augmented with the original dataset. Here, we used two techniques to generate the synthetic images: geometric transformation and brightness adjustment. The overall summary of images in the MDSD database is provided in **Table 2**.

#### 3.5.1 Geometric transformation

Geometric transformation means transforming the orientation of the images. In this study, we applied several geometric transformations randomly to generate artificial images which involved rotating (90°, 180° and 270°), flipping (top-down and left-right), skewing, and zooming. The geometric transformations were applied using the ‘Augmentor’ library (Bloice et al., 2019) which provides translation invariance transformation of the images.

#### 3.5.2 Brightness adjustment

As the images were captured using different devices and at different periods of time, the images weren’t homogeneous in terms of illumination. The light intensity on the diseased images greatly impacts when we apply computer vision techniques. Hence, we applied a *gamma function* in our images to generate synthetic images with different brightness levels. The *gamma function* is an image processing technique that applies the non-linear adjustment to individual pixel values to encode and decode the luminance of an image. The gamma function can be defined mathematically by the following formula (eq. 1).


$$i_{out}=ai_{in}^{\left(1/\gamma \right)}$$

where, *i_in_* is the input images with pixel values scaled from [0, 255] to [0, 1], *γ* is the gamma value, *i_out_* is the output image scaled back to [0, 255] and *a* is a constant value (mainly equal to 1). The gamma values ( *γ* ) < 1 will shift the image towards the darker end of the spectrum while gamma values ( *γ* ) > 1 will make the image brighter and the *γ*=1 will not affect the input image.”

### 3.6 Proposed lightweight CNN model

In this study, we proposed a lightweight convolutional neural network (CNN) to identify the severity stages of MLB disease of maize crop. In this network, we have incorporated the modified *Inception* modules into a simple CNN framework, enhancing the network’s finer and multi-scale feature extraction capability. The proposed model is composed of several computational modules which are discussed in following subsections:

#### 3.6.1 CRB layer (*crb*)

**Figure 3:**
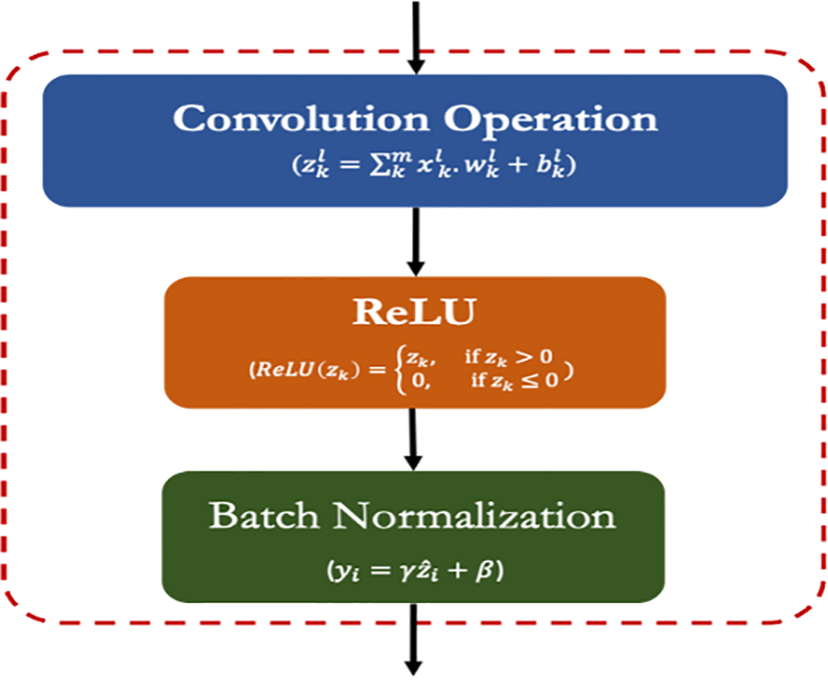
Framework of the CRB module of the proposed model.

The CRB is the most important layer in the proposed lightweight model which encompasses three popular operations viz. Convolution operation, ReLU and Batch Normalization operation as shown in **Figure 3**. The main function of this CRB layer was to generate pattern detectors from the images in the form of feature maps.

##### 3.6.1.1 Convolution operation (conv)

The convolution operation involves the extraction of inherent features (aka feature maps) from the input images by using a set of kernels/filters (LeCun et al., 1998). The kernel/filters are of smaller size than the input images such as 3 × 3 or 1 × 1. Mathematically, the convolution operation is expressed by eq. 2:


$$z_{k}^{l}=\sum_{k}^{m}x_{\;k}^{l}.w_{k}^{l}+b_{k}^{l}$$

where,

$z_{k}^{l}$ denotes the output feature map of *k*-th input at *l*-th layer of the model

$x_{k}^{l}$ denotes the *k*-th input feature map at *l*-th layer of the model

$w_{k}^{l}$ and $b_{k}^{l}$ denotes the weights and bias at the *l*-th layer of the model

##### 3.6.1.2 ReLU operation (ReLU)

ReLU (Rectifier Linear Unit) is the widely used activation function for the CNN models that enhances the non-linear attributes within the input feature maps (Haque et al., 2021). The ReLU function requires less computation hence speed up the overall training process. Its convergence speed is higher than the other functions and induces sparsity in feature maps. It is expressed by the following equation (eq 3):


$$ReLU\left(z_{k}\right)=\{\begin{array}{c} z_{k},\;\;\;\text{if}\;z_{k}\;> 0\;\\ 0,\;\;\;\;\text{if}\;z_{k}\;\leq 0 \end{array}$$

where, *z_k_* denotes the output feature map of *k*-th input feature map

##### 3.6.1.3 Batch normalization operation (BN)

The batch normalization process transforms a batch of images (say *m*) to have a mean zero and standard deviation of one. It speeds up the training process and handles the internal covariances of the input feature maps (Ioffe, 2017). The batch normalization is expressed as the following equations (eq. 4 and eq. 5):


$$y_{i}=\gamma {\hat{z}}_{i}+\beta \;$$

$${\hat{z}}_{i}=\frac{z_{i}-E(z_{i})}{\sqrt{var\left(z_{i}\right)+\epsilon }}$$

where,

*y*_*i*_ denotes the output feature map

${\hat{z}}_{i}$ is the normalized input feature map

*E*(*z*_*i*_) denotes the mean of the input feature map *z*_*i*_ *var*(*z*_*i*_) denotes the variance of the input feature map *z*_*i*_ *γ* *and* *β* are the scaling and offset factors of the network that are trainable

#### 3.6.2 Maxpool module (*pool*)

The maxpooling operation extracts the maximum element from the respective regions of feature map covered by the pooling kernels (Chollet, 2021). The maxpool layer outputs the most promising features from the input images without adding any extra trainable parameters to the network. In this proposed model, we applied maxpool with a kernel size of 3 x 3 and strides of 1 and 2.

#### 3.6.3 Modified inception module (*incep*)

**Figure 4:**
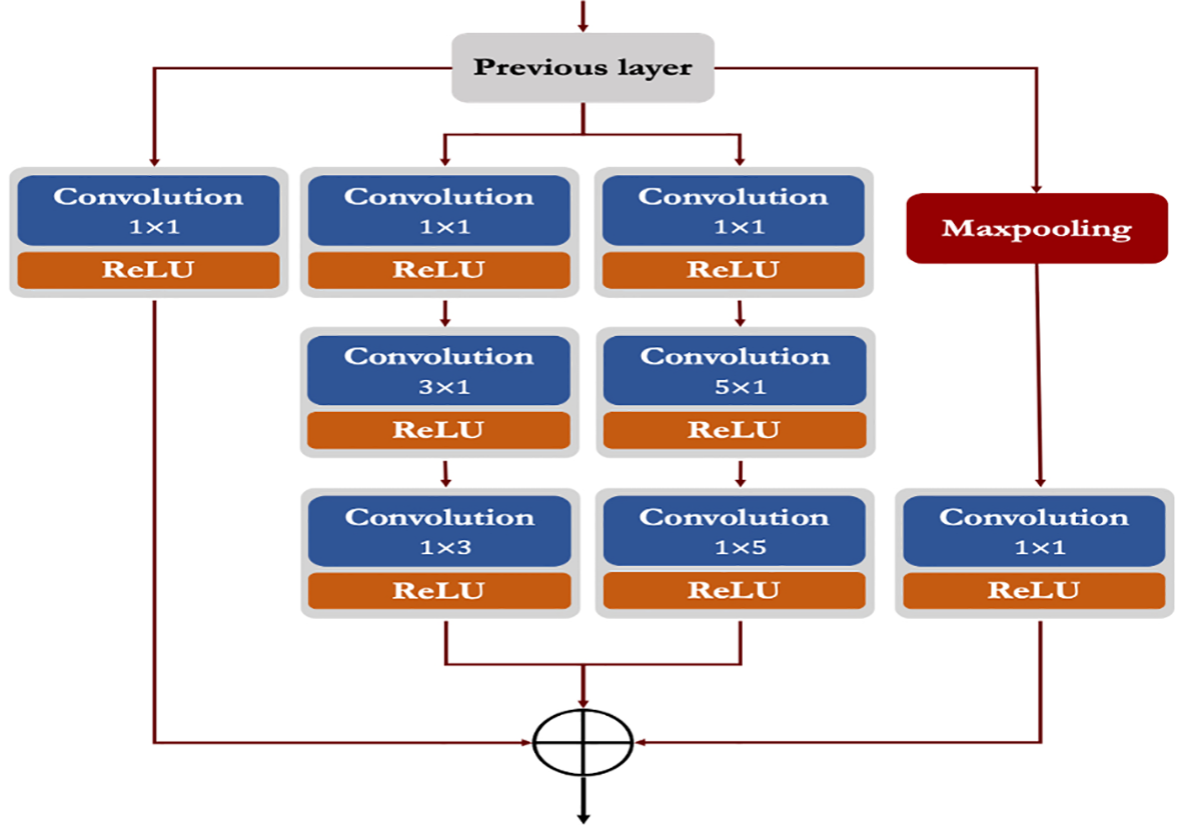
Architecture of the proposed modified inception module.

Generally, the ‘inception’ module of Inception networks obtain the integration of sparse structure by approximating the available dense component of the network (Szegedy et al., 2015; Szegedy et al., 2016). In this study, we proposed a modified inception module by applying few changes with respect to the kernel sizes, number of filters and parallel convolutions. In the proposed inception module, we applied symmetrical (1 x 1) and asymmetrical convolution kernels in a parallel manner with a maxpool operation. Here, we factorized the convolutions with spatial filters of n × n (for 3 x 3 or 5 x 5) into asymmetrical convolutions with filter sizes n×1 and 1×n (e.g., 3 x 1 and 1 x 3; 5 x 1 and 1 x 5). Prior to each asymmetrical convolution, one 1 x 1 convolution kernel is incorporated to reduce the representational bottleneck of the network. We also applied ReLU in each convolution operation to induce sparsity in the feature maps (as shown in **Figure 4**). Finally, the outputs from all parallel convolutions and maxpool layers were concatenated and passed to the next layer of the network.

#### 3.6.4 GAP module (*gap*)

The GAP or Global Average Pooling is a unique pooling operation designed to generate a scalar vector of features by computing the average of each feature map. It aggressively summarizes the presence of a feature in an image by downsampling the entire input feature map to a single value (Lin et al., 2013). The purpose of the GAP layer was to reduce the chance of overfitting as it doesn’t add any extra learnable parameters to the network.

#### 3.6.5 Softmax layer (*softmax*)

A softmax layer was added at the end point of the proposed CNN model. The softmax layer contains the same number of nodes as the number of classes in the dataset under study. The *softmax function* generates the output probability values from the input feature vectors. It converts the non-normalized feature vectors of the network into a probability distribution over the predicted output class (Bouchard, 2007). Mathematically, softmax function is expressed as the following equation (eq: 6):


$$Softmax\left(z_{j}\right)=\frac{e^{z_{j}}}{\sum_{j}e^{z_{j}}}$$

where, *z_j_* denotes the *j*-th item of the output feature vector

**Figure 5:**
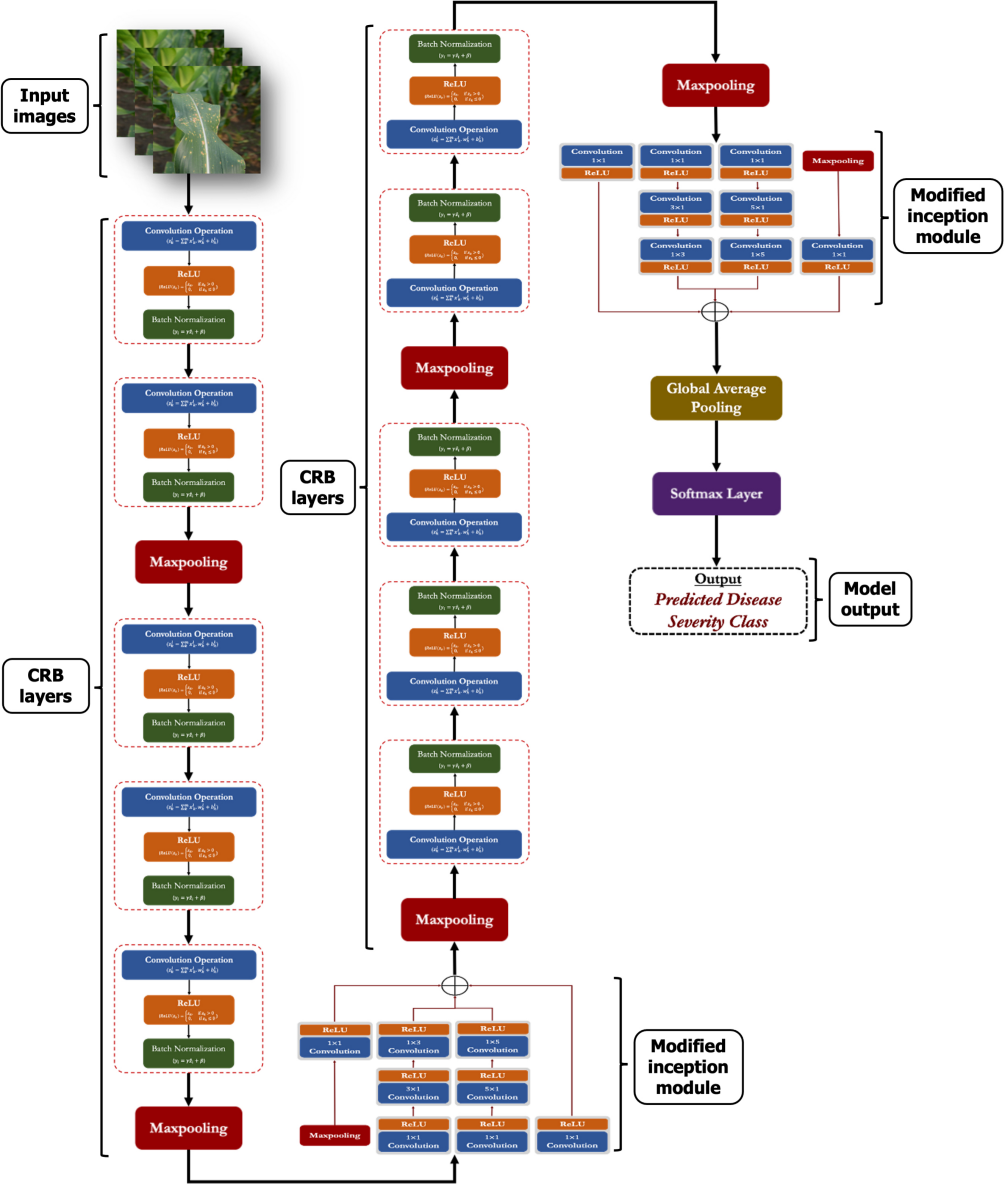
Overall architectural framework of the proposed CNN model.

The overall framework of the proposed network in a graphical manner is provided in **Figure 5**. Also, a detailed layer-wise description like layer names, kernel/filter sizes, strides, output shapes, number of kernels/filters and number of training parameters is provided in **Table 3**.

**Table 3:** Layer-wise configuration of the proposed model.

Name	Layers	Kernel size	Stride	Output shape	# Kernel	Parameters
***input***	Input images	–	–	256 x 256 x 3	–	0
***crb_1***	Conv + ReLU + BN	3 x 3	1	256 x 256	32	864 + 96 + 0
***crb_2***	Conv + ReLU + BN	3 x 3	1	254 x 254	64	18,432 +192 + 0
***pool_1***	Max-Pooling	3 x 3	2	126 x 126	64	0
***crb_3***	Conv + ReLU + BN	3 x 3	1	126 x 126	64	36,864 + 192 + 0
***crb_4***	Conv + ReLU + BN	1 x 1	1	126 x 126	96	6,144 + 288 + 0
***crb_5***	Conv + ReLU + BN	1 x 1	1	126 x 126	96	9,216 + 288 + 0
***pool_2***	Max-Pooling	3 x 3	1	124 x 124	96	0
***incep_1***	Inception	1 x 1, 3 x 1, 1 x 3, 5 x 1, 1 x 5	1	124 x 124	32, 64, 128, 256	72,128
***pool_3***	Max-Pooling	3 x 3	2	61 x 61	256	0
***crb_6***	Conv + ReLU + BN	1 x 1	1	61 x 61	128	32,768 + 384 + 0
***crb_7***	Conv + ReLU + BN	3 x 3	1	61 x 61	128	1,47,656 + 384 + 0
***crb_8***	Conv + ReLU + BN	1 x 1	1	61 x 61	256	32,768 + 768 + 0
***pool_4***	Max-Pooling	3 x 3	1	59 x 59	256	0
***crb_9***	Conv + ReLU + BN	1 x 1	1	59 x 59	256	65,536 + 768 + 0
***crb_10***	Conv + ReLU + BN	3 x 3	1	59 x 59	256	8,84,736 + 1152 + 0
***pool_5***	Max-Pooling	3 x 3	2	29 x 29	384	0
***incep_2***	Inception	1 x 1, 3 x 1, 1 x 3, 5 x 1, 1 x 5	1	29 x 29	32, 64, 128, 256	1,94,008
***gap***	Global average pooling	–	–	320		0
***softmax***	Softmax layer	–	–	1 x 4		1,248

### 3.7 Evaluation metrics

We evaluated the prediction performance of the proposed CNN model on the images of testing data. We computed the confusion matrix (*CM*) which represents the model’s prediction performance in a tabular fashion. In *CM*, row elements denote the actual values, while the column entities present the predicted values. In the *CM* the diagonal elements represent the correct predictions (i.e. true positives (TP) and true negatives (TN)), while the incorrect predictions (i.e. false positives (FP), false negatives (FN)) are denoted by the off-diagonal elements. Also computed the relevant evaluation metrics such as Recall, Precision and f1-score.

**Classification Accuracy:** The classification accuracy (or accuracy) defines the proportion of the correct prediction out of the total predictions. The following expression measures it:


$$\text{C}lassification\;Acuracy=\;\frac{\left(True\;Positives\;\left(TP\right)+True\;Negeatives\;\left(TN\right)\right)}{\left(Number\;of\;samples\;in\;the\;dataset\right)}$$

**Recall (Sensitivity):** The recall or sensitivity is the measure which tells that the % of actual positive are predicted as positive. The following expression calculates it-


$$Recall\;\left(Sensitivity\right)=\;\frac{\left(True\;Positives\;\left(TP\right)\right)}{\left(\;False\;Negeatives\left(FN\right)+\;True\;Postives\;\left(TP\right)\right)}$$

**Precision:** Precision is the measure which gives the % of predicted as positives that are actually positive. The following expression calculates it-


$$\text{P}recision=\;\frac{\left(True\;Positives\;\left(TP\right)\right)}{\left(False\;positives\;\left(FP\right)\;+\;True\;Positives\;\left(TP\right)\right)}$$

**f1-Score**: f1-Score is the measure that tells us about the robustness of the model. It is the harmonic mean of precision and recall. The following expression calculates it-


$$f1-Score=\;2*\frac{\left(Precision*Recall\right)}{\left(Precision+Recall\right)}$$

## 4 Results and discussion

In this study, 1,760 images of MLB disease of maize were collected under the MDSD database from agricultural fields which were then augmented to 14,389 images. The MDSD image database is categorized into 4 groups viz. healthy, low severity, medium severity and high severity based on the intensity levels of the disease symptoms on leaves. We randomly split the whole dataset into two sets viz. training and testing sets in the ratio of 80:20. Here, the proposed convolutional neural network (CNN) was trained and tested with the MDSD dataset for automated diagnosis of severity stages of MLB disease. In this approach, several combinations of *CRB* and inception modules were attempted. However, CNN network with 10 *CRB* and 2 modified inception modules gave the optimal classification performance. Furthermore, to inspect the effectiveness of the proposed model, we also employed a few state-of-the-art pre-trained models viz: VGG16, VGG19, InceptionV3, ResNet50, Xception, MobileNetV2, DenseNet121 and NASNetMobile networks in this study. All the models were trained and tested with similar hyperparameters and configurations as shown in **Table 4**. All the model architectures were implemented in python using the tensorflow environment, an open-source deep learning framework. We performed all the experimental analyses by utilizing the high computation power of the Tesla V100 GPUs in the NVIDIA DGX servers.

**Table 4:** Hyperparameters used during model training.

Name	Hyper Parameters
Loss Function	Categorical Cross Entropy
Optimization function	Nadam
Learning Rate	0.001
Momentum	0.9
Weight Decay	0.004
Epochs	500
Batch size	128

**Figure 6:**
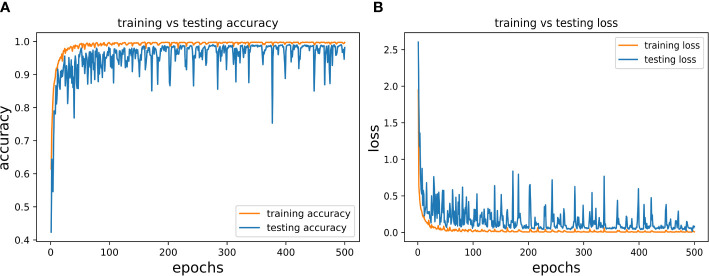
Epoch-wise behaviour of training and testing of the proposed CNN model **(A)** Classification accuracy: training vs testing and **(B)** Loss: training vs testing.

In the present study, we trained and validated our proposed CNN model 500 times (epochs) using a batch size of 128 (according to the hardware system feasibility) on the MDSD database. Our proposed model achieved the training accuracy of 99.78% with loss of 0.046, whereas the testing accuracy achieved so far was 99.13% with loss of 0.0317. We presented the epoch-wise training and testing behavior (for both classification accuracy and loss) of the proposed model in **Figures 6A, B** to showcase the model’s efficiency on images of MDSD database.

**Figure 7:**
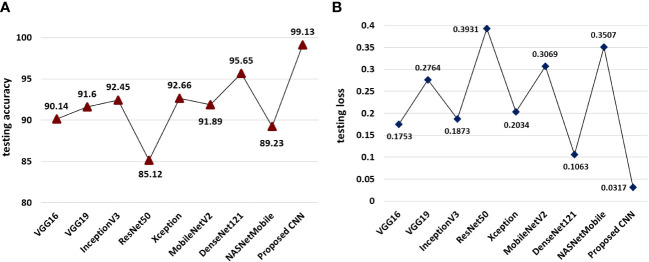
Comparative performance of the proposed model and pretrained models **(A)** models wise classification accuracies on test data and **(B)** model-wise testing loss.

The experimental findings on the testing set of the MDSD image database reported that our proposed model achieved the overall classification accuracy (99.13%) which is far better than the employed pre-trained networks as shown in **Figures 7A, B**. However, among the state-of-the-art pre-trained models, the DenseNet121 model achieves the highest accuracy of 95.65% on the test dataset (shown in **Figure 7A**). The rest of the models achieve accuracy within 85 to 92%. The proposed model also obtained the lowest (0.0317) of all, while the DenseNet121 model reaches 0.1063 (can be seen in **Figure 7B**). These experimental results cater the superiority and effectiveness of the proposed model over the popular pre-trained models.

**Figure 8:**
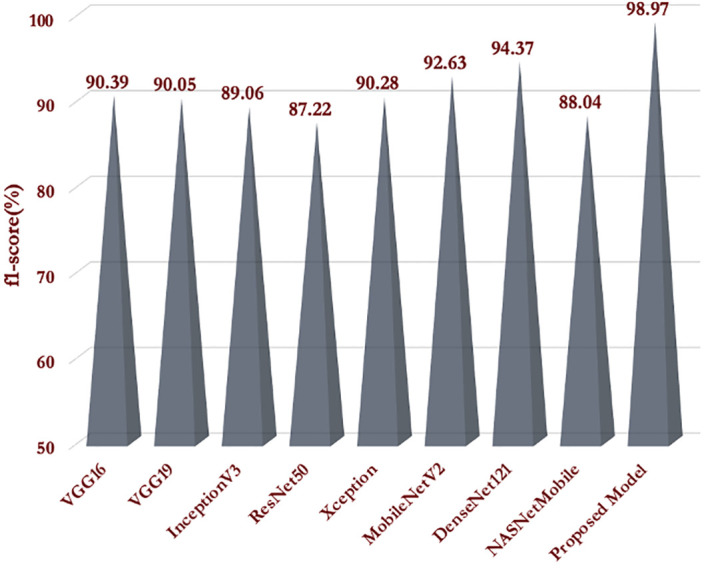
f1-scores of the models obtained on testing dataset.

The interpretation of the model’s performance evaluation based on classification accuracy and training loss wouldn’t be sufficient. Hence, we calculated the average f1-scores of all the models to evaluate the models in an unbiased way. We presented the obtained f1-scores of the models (proposed as well as pre-trained) in **Figure 8**. It is quite evident from **Figure 8**, that our proposed model obtained the highest f1-score than the pre-trained models in the testing dataset of MLB disease. Our proposed model’s prediction performance on the MLB disease dataset was far better than the popular pretrained models. This result implies that our proposed CNN model could identify the unknown images of MDSD database and classify them into respective severity classes.

**Figure 9:**
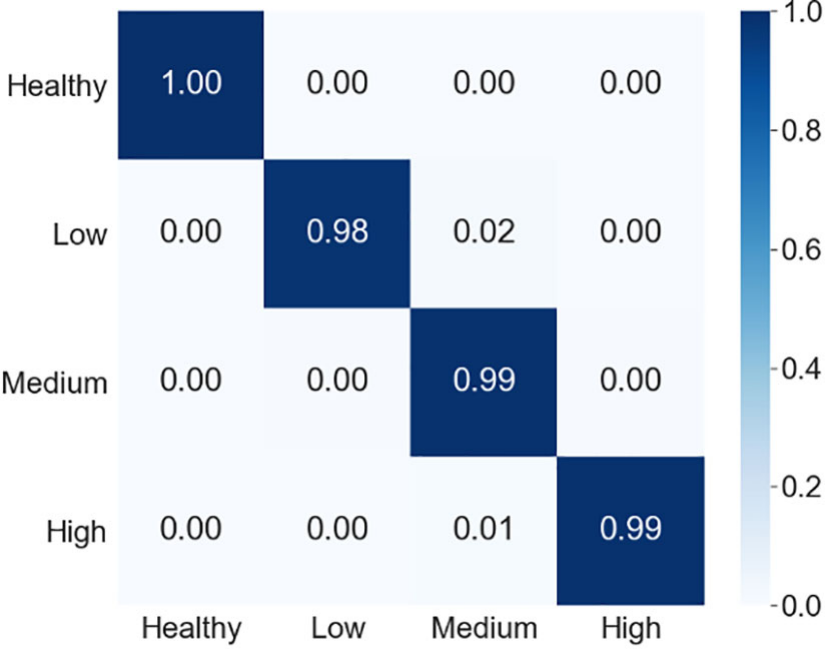
Confusion matrix of the proposed model on testing dataset.

To better understand the prediction performance of our proposed model, we presented the confusion matrix in **Figure 9**. **Figure 9** shows that our proposed model was 100% accurate in predicting the healthy samples, 98% accurate for the low severity samples, 99% accurate for both samples of medium severity and high severity. Moreover, we also computed recall, precision and f1-score to present the class-wise prediction performance of the proposed model as shown in **Table 5**. **Table 5** shows that the proposed model obtained quite high scores (approx. 99%) for all three metrics. It is evident from the confusion matrix and the performance metrics (recall, precision, and f1-score) that our model performed remarkably well for all the classes of the severity of MLB disease in MDSD database. The model’s performance was quite appreciable not only for healthy or high severity images but also for low severity images in which the symptoms of the disease are very mild. This result supports the significance of the proposed CNN model in recognizing severity levels for the unknown images of MLB disease of maize crop.

**Table 5:** Class-wise performance of the proposed model.

Category	Recall (%)	Precision (%)	f1-score (%)
**Healthy**	100	100	100
**Low Severity**	99.20	98.02	98.61
**Medium Severity**	98.13	98.55	98.34
**High Severity**	98.60	99.30	98.95
**Average**	98.98	98.97	**98.97**

From the overall analysis of all the employed models, it is apparent that our proposed lightweight CNN model outperforms the popular pre-trained models for identifying the severity stages of MLB disease. However, the most important aspect of this study is that the proposed model can identify the images of the severity of MLB disease even with complex background conditions. This makes the proposed CNN model an effective and cost-effective approach for identifying the appropriate disease severity stages for the researchers, subject matter specialists and farmers in the field condition.

## 5 Ablation studies

**Figure 10:**
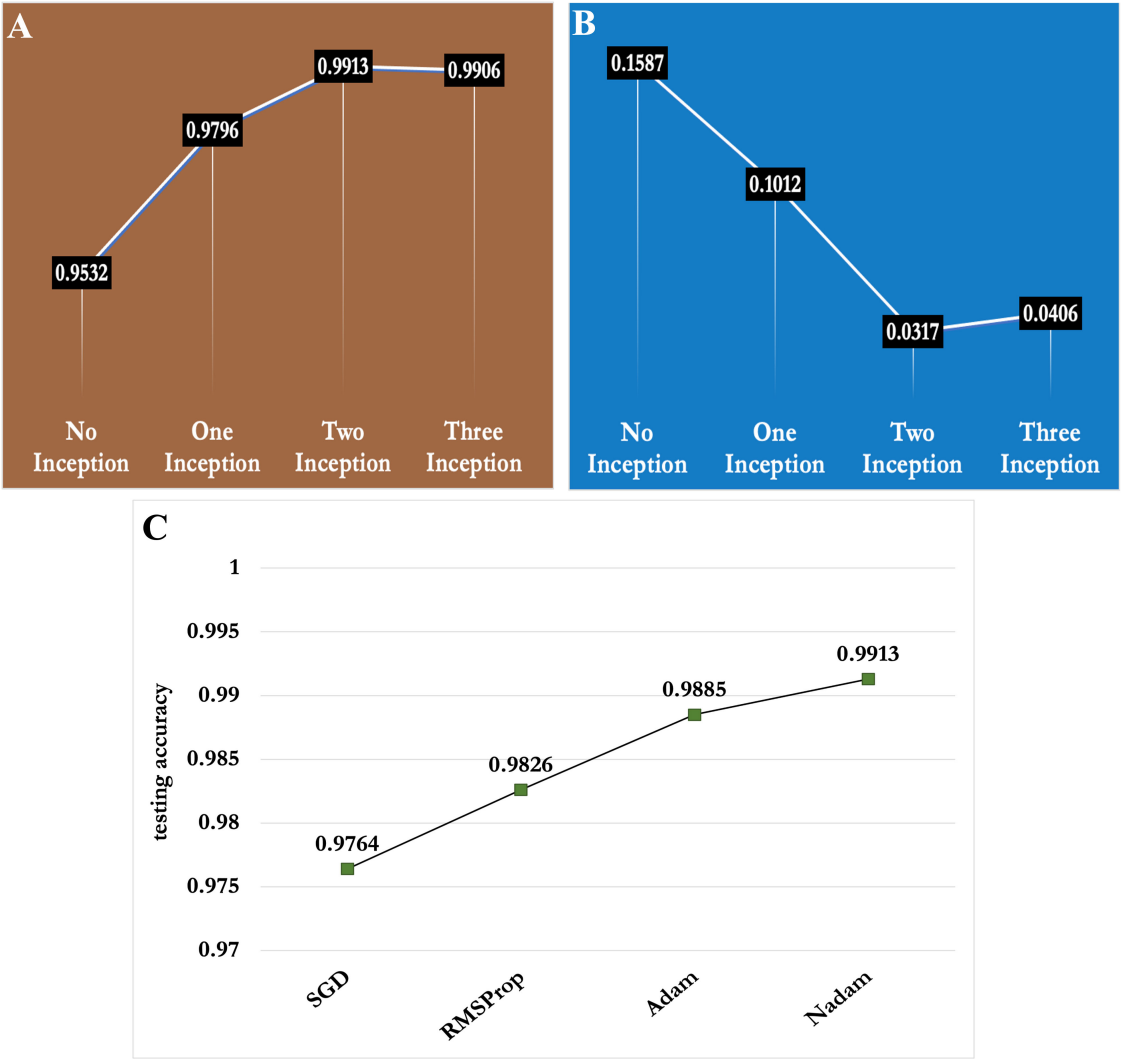
Depiction of the effect of number of *Inception* modules and optimization functions in performance of the proposed CNN model **(A)** Number of Inception modules vs classification accuracy **(B)** Number of Inception modules vs testing loss and **(C)** Optimization functions vs classification accuracy .

In this section, we presented the ablation studies for selecting the optimum number of inception modules and best optimization function for the proposed model. First, we trained our proposed CNN model by incorporating 0,1,2 and 3 *Inception* modules. The experimental results reported in **Figures 10A, B**, depict that the proposed CNN framework achieved around 95% testing accuracy without any inception module. However, the accuracy kept increasing as the number of Inception modules increased as shown in **Figure 10A**. As a result, the proposed model showed the best prediction performance (classification accuracy of 99.13%) with two *Inception* modules compared to the others. From **Figure 10B**, it is apparent that as the number of *Inception* modules increased, the testing loss decreased and the proposed model achieved the lowest testing loss (i.e. 0.317) with two *Inception* modules. Hence, the two Inception modules were selected for the proposed CNN model.

We also conducted experiments with different optimization functions, which have a huge role in model convergence and feature learning. We experimented with four types of optimization functions viz. *Stochastic gradient descent* (*SGD*), *RMSProp*, *Adam* and *Nadam* in the proposed model and presented the results in **Figure 10C**. Among the four optimization functions, *Nadam* function showed the best performance in the MLB disease severity dataset of maize crop.

## 6 Conclusion

In this study, we addressed the major issue of crop management i.e., disease severity stages by proposing a deep learning-based diagnosis approach. In this regard, we created an image database known as MDSD containing images of MLB disease with four different severity stages viz. healthy, low severity, medium and high severity. Next, we proposed a novel lightweight CNN model to identify of severity stages of MLB disease using the images of MDSD. The proposed CNN model’s basic framework comprises a stack of computational layers like the CBR layer (*Convolution, ReLU and Batch normalization*) augmented with two modified *Inception* modules. On the test dataset, our proposed model reported 99.13% classification accuracy with an f1-score of 98.97% which is quite superior than most of the popular state-of-the-art pretrained models. Furthermore, the overall experimental analysis demonstrated that our proposed CNN model efficiently captures the promising features of the images with complex backgrounds and classifies them into respective severity classes. Therefore, this automated approach for identifying the severity stages of MLB disease using the proposed CNN model would be feasible and cost-effective for the farm community and the subject matter specialists. However, in the present study, the proposed CNN model only applies to the MLB disease of maize crop. In the future, the study can be further expanded to identify severity stages of other major diseases of maize crop and diseases of other crops as per the availability of image dataset.

## Data availability statement

The raw data supporting the conclusions of this article will be made available by the authors, without undue reservation.

## Author contributions

MH: Conceptualization, Methodology, Investigation, Writing - Original Draft, Visualization; SM: Conceptualization, Supervision, Investigation; AA: Supervision, Writing - Review and Editing; CD: Conceptualization, Supervision, Writing - Original Draft, Visualization; TM: Writing - Review and Editing; SN: Writing - Original Draft, Visualization; KH: Field data generation; Data curation. All authors contributed to the article and approved the submitted version.
